# Pharmacoethnicity of FOLFIRINOX versus gemcitabine plus nab-paclitaxel in metastatic pancreatic cancer: a systematic review and meta-analysis

**DOI:** 10.1038/s41598-021-99647-5

**Published:** 2021-10-11

**Authors:** Yoon Suk Lee, Jong-chan Lee, Jae-Hyeong Kim, Jaihwan Kim, Jin-Hyeok Hwang

**Affiliations:** 1 Department of Internal Medicine, Ilsan Paik Hospital, Inje University College of Medicine, Goyang, Republic of Korea; 2 Department of Internal Medicine, Seoul National University College of Medicine, Seoul National University Bundang Hospital, 82, Gumi-ro 173 Beon-gil, Bundang-gu, Seongnam, 13620 Republic of Korea

**Keywords:** Gastroenterology, Oncology

## Abstract

Treatment outcomes between FOLFIRINOX (5-fluorouracil, leucovorin, irinotecan, and oxaliplatin) and GNP (gemcitabine with albumin-bound paclitaxel) as first-line chemotherapy regimens for metastatic pancreatic cancer (PC) were assessed according to ethnic groups categorized as Western or Asian subgroups. PubMed, EMBASE, and Cochrane library were searched. Thirteen studies were eligible in this meta-analysis. Overall survival was not significantly different between FOLFIRINOX and GNP (HR 1.00, 95% CI 0.83–1.20, *P* = 0.990). However, the Western subgroup showed a higher survival benefit for FOLFIRINOX over GNP (HR 0.84, 95% CI 0.74–0.95, *P* = 0.006) whereas the Asian subgroup showed the survival benefit for GNP over FOLFIRINOX (HR 1.29, 95% CI 1.03–1.60, *P* = 0.030). Progression free survival was not significantly different between the two regimens in the Western subgroup (HR 1.01, 95% CI 0.84–1.20, *P* = 0.950) and the Asian subgroup (HR 1.13, 95% CI 0.97–1.33, *P* = 0.110). Occurrence of febrile neutropenia was significantly higher in FOLFIRINOX at both ethnic subgroups; however, that of peripheral neuropathy was significantly higher only in GNP of the Asian subgroup. Therefore, pharmacoethnicity might be a factor worth considering when deciding on a frontline chemotherapeutic regimen although the overall survival was not significantly different between FOLFIRINOX and GNP for metastatic PCs.

## Introduction

Pancreatic cancer (PC) is well known for its dismal prognosis with an overall survival (OS) between 5 and 8%, despite advancements in novel chemotherapeutics and targeted agents^1^. Since the introduction of gemcitabine, various combinations of different anticancer drugs have been studied to demonstrate their survival benefit in PC. However, compared with the original gemcitabine monotherapy, only FOLFIRINOX (5-fluorouracil, leucovorin/folinic acid, irinotecan, and oxaliplatin) and gemcitabine with albumin-bound paclitaxel (GNP) have shown clinically significant survival benefit in patients with metastatic PCs^2–4^. Thus, these two regimens are recommended as first-line treatments for metastatic PC^5,6^. However, the superiority of one regimen over another has not been fully investigated; randomized prospective trials comparing the benefits of FOLFIRINOX and GNP in patients with metastatic PCs have not been conducted, thereby making it difficult to draw a conclusion as to which one of these should be in the first choice among the first-line regimens.

Recently, the ethnic diversity, which accounts for inter-individual variations in drug responses or toxicities, has been increasingly recognized as an important factor in cancer therapeutics^7–10^. Furthermore, while the ethnic differences in the chemotherapeutic responses between Western and Asian patients has been reported in several studies^11–13^, to the best of our knowledge, ethnic differences influencing the treatment outcomes of FOLFIRINOX and GNP in metastatic PCs have not been studied so far. Therefore, this meta-analysis aimed to compare treatment outcomes between FOLFIRINOX and GNP as first-line chemotherapy for patients with metastatic PCs and to figure out its differences based on the ethnic groups of patients, categorized into Western and Asian subgroups.

## Methods

### Search strategy and selection criteria

This systematic review and meta-analysis followed the guidelines of the Preferred Reporting Items for Systematic Reviews and Meta-Analyses^14^. We searched PubMed, EMBASE and Cochrane Library from the first of January 2009 to the end of March 2020 for eligible studies comparing survival outcomes between FOLFIRINOX and GNP for patients with metastatic PC as a first-line palliative chemotherapy. Search strategies of each electronic database are summarized in Supplementary Table S1. In addition, a manual search of the bibliographies of included studies was conducted.

The inclusion criteria were as follows: (1) populations: patients with metastatic PCs; studies which enrolled locally advanced PCs were included if the studies separately reported survival outcomes between locally advanced and metastatic PCs. (2) intervention: first-line systemic chemotherapy with FOLFIRINOX; (3) comparison: first-line systemic chemotherapy with GNP; (4) primary outcome: OS, secondary outcomes: progression free survival (PFS) and chemotherapy-related adverse events; for the chemotherapy-related adverse events, studies which enrolled locally advanced PCs as well as metastatic PCs were also included if the study reported toxicity outcomes. (5) study design: prospective or retrospective studies, and nationwide population studies were also eligible if they investigated the survival outcomes. To be eligible for this meta-analysis, the studies should provide hazard ratios (HR) or crude data, and the corresponding standard errors (SE), variance, 95% confidence interval (CI), or *P* value. Otherwise, the articles had to provide the Kaplan–Meier survival curves with the total numbers in each group for the estimation of HR and 95% CI.

Using a data extraction form, two investigators (YS Lee and JC Lee) independently reviewed and extracted information on the first authors, years of publication, participating institutions, countries in which the studies were conducted, study designs, study durations, inclusion and exclusion criteria, numbers of study participants, chemotherapy regimens, survival outcomes (OS and PFS), chemotherapy-related toxicities (anemia, thrombocytopenia, neutropenia, febrile neutropenia, peripheral neuropathy), and analysis types. In terms of the ethnicities, the countries where the study was conducted were regarded as the representative of ethnicity and then categorized into either the Western or Asian subgroups.

### Data analysis

Direct meta-analysis was performed to calculate pooled HRs with 95% CIs for each comparison between the chemotherapeutic regimens using a random-effects model. The HRs with 95% CIs were extracted from the reported data. However, if the HRs could not be directly extracted, they were estimated using the method described by Parmar et al.^15^ or calculated from Kaplan–Meier survival curves using that of Tierney et al.^16^. The Chi-square-based Q-test and I^2^ statistics test were used to evaluate the heterogeneity of included studies. The heterogeneity was considered statistically significant if *P*-value was < 0.1 or the I^2^ statistic was > 50%^17^, and was evaluated using sensitivity analyses. A funnel plot was constructed to assess for any publication bias. Direct meta-analyses were performed with meta-analysis software RevMan (version 5.3, Nordic Cochrane Center, Copenhagen, Denmark).

The Risk of Bias Assessment Tool for Nonrandomized Studies (RoBANS) was used to evaluate the quality of the enrolled studies. RoBANS has been well validated as a quality assessment tool for retrospective and non-randomized studies^18^.

## Results

**Figure 1:**
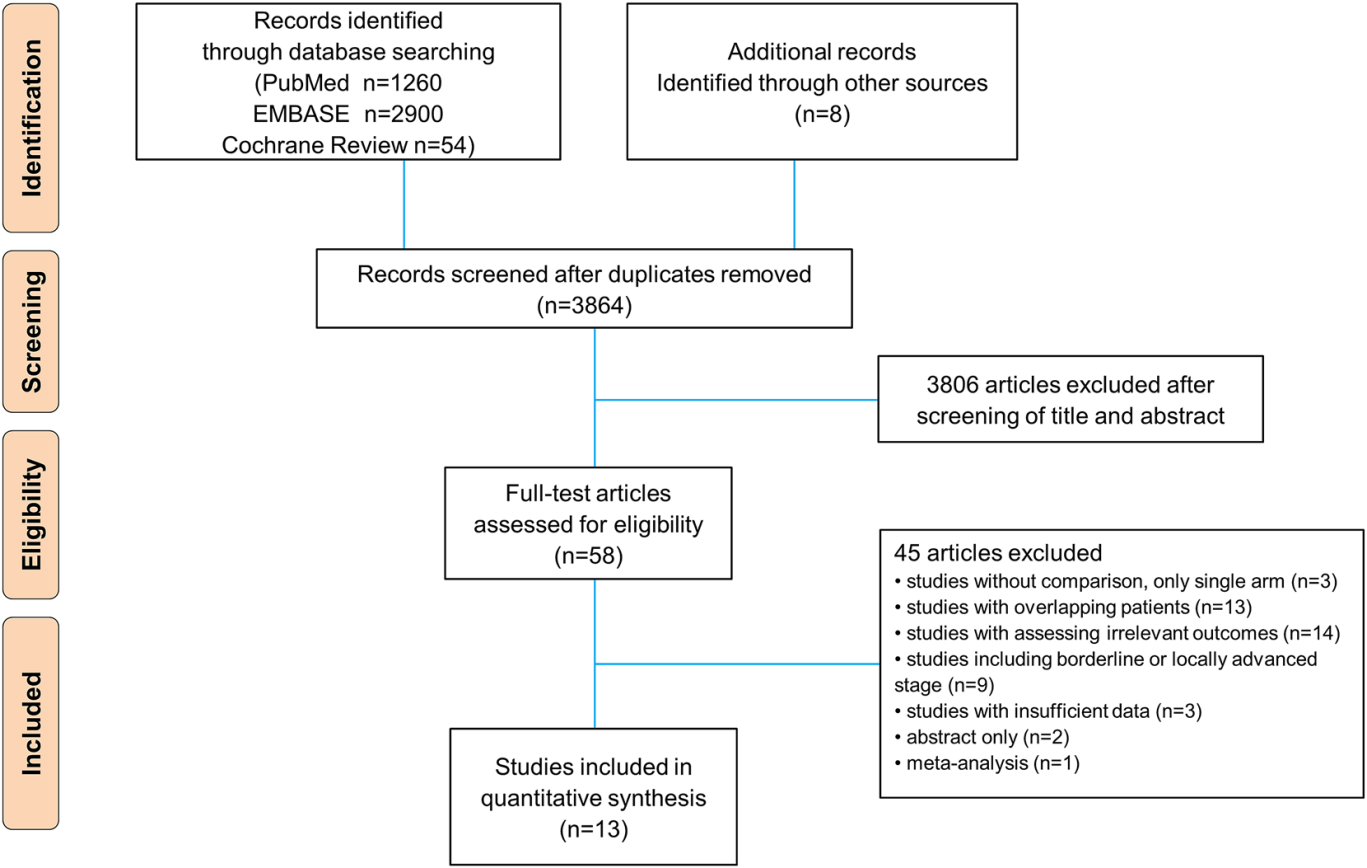
PRISMA flow chart.

A flow diagram of our systematic review is shown in Fig. 1. The thirteen studies finally enrolled in this meta-analysis comprised 1952 patients treated with FOLFIRINOX and 1,889 patients treated with GNP. Among the 13 studies included, 11 studies were two-arm study design for comparing FOLFIRINOX versus GNP^19–29^, and the other two studies were designed as a three-arm study that compared the efficacy between FOLFIRINOX, GNP, and gemcitabine monotherapy^30,31^. Eight studies enrolled only patients with metastatic PCs^19–21,23,25,26,29,30^, whereas the other five studies included patients with locally advanced PCs as well as metastatic PCs^22,24,27,28,31^. Eight studies were conducted in Western countries, and five were conducted in Asian countries. The main characteristics of the enrolled studies are summarized in Supplementary Table S2. Regarding age and performance status, the patients treated with FOLFIRINOX were younger and had a better performance status than those with GNP (Supplementary Figs. S1, S2). These findings were significant for the Western subgroup, while for the Asian subgroup, they were not. Their quality assessments are summarized in Supplementary Fig. S3.

**Figure 2:**
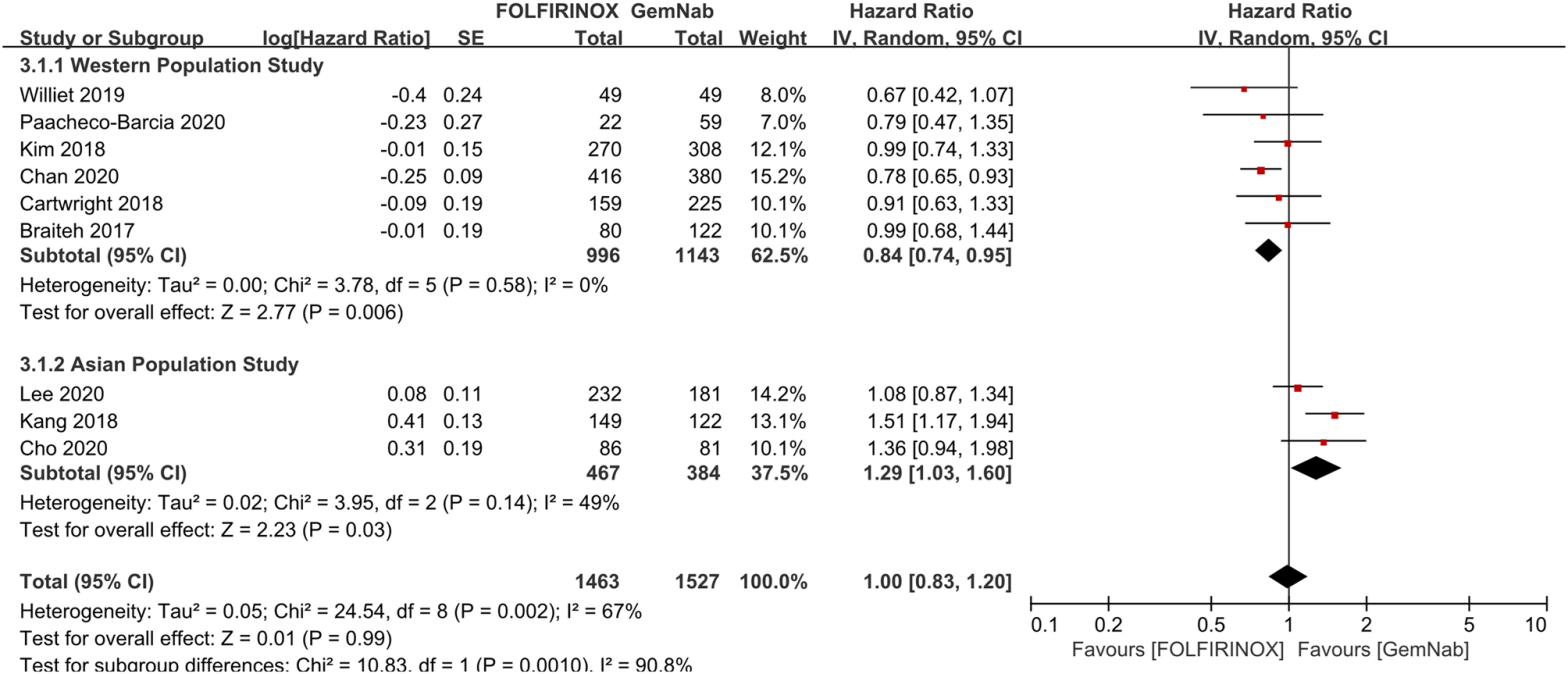
The pooled results of overall survival between FOLFIRINOX and GNP using random effects model did not show the survival difference. However, subgroup analyses according to the ethnicity showed that Western subgroup showed the survival benefit of FOLFIRINOX (HR 0.84, 95% CI 0.74–0.95, *P* = 0.006); Asian subgroup showed opposite results (HR 1.29, 95% CI 1.03–1.60, *P* = 0.030).

For the OS in patients with metastatic PCs, nine studies with 1463 patients with FOLFIRINOX and 1527 patients with GNP were included. The pooled meta-analysis showed that there was no significant difference (HR 1.00, 95% CI 0.83–1.20, *P* = 0.990) (Fig. 2). However, it showed a significant heterogeneity (I^2^ = 67%). Subgroup analyses were conducted to evaluate the effects between the Western and Asian subgroups. The Western subgroup showed a higher survival benefit for FOLFIRINOX (HR 0.84, 95% CI 0.74–0.95, *P* = 0.006). Conversely, the Asian subgroup showed opposite results (HR 1.29, 95% CI 1.03–1.60, *P* = 0.030), revealing the survival benefit for GNP. Furthermore, the heterogeneity turned out to be insignificant within each subgroup (Western subgroup: *P* = 0.580, I^2^ = 0%; Asian subgroup: *P* = 0.140, I^2^ = 49%).

**Figure 3:**
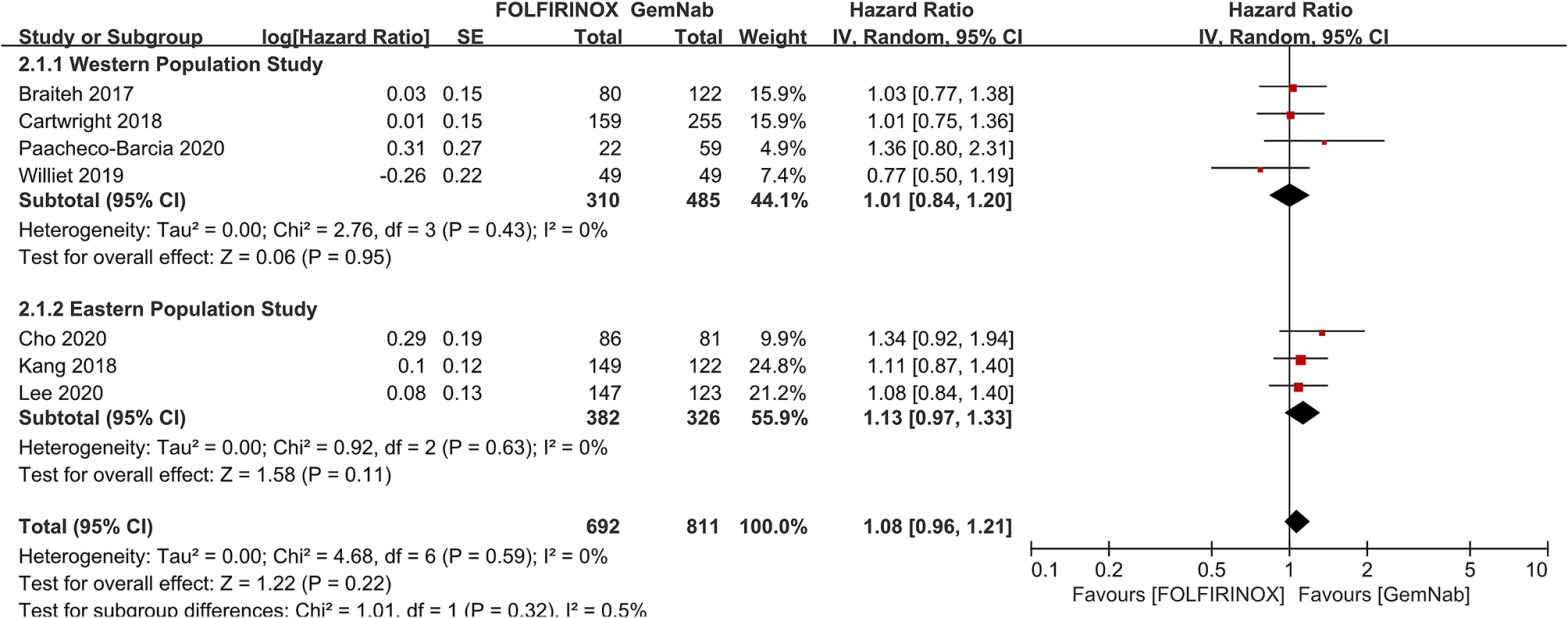
The pooled results of progress free survival between FOLFIRINOX and GNP using random effects model did not show the survival difference. Subgroup analyses of PFS did not show significant difference between Western (HR 1.01, 95% CI 0.84–1.20, *P* = 0.950) and Asian subgroups (HR 1.13, 95% CI 0.97–1.33, *P* = 0.110). However, it revealed that Asian subgroup showed the trends of survival benefit for GNP.

For the PFS in patients with metastatic PCs, seven studies with 692 patients with FOLFIRINOX and 811 patients with GNP were included. The pooled results of the seven studies showed that there was no significant difference (HR 1.08, 95% CI 0.96–1.21, *P* = 0.220). Unlike the results of OS, the subgroup analyses of PFS did not show significant difference between Western (HR 1.01, 95% CI 0.84–1.20, *P* = 0.950) and Asian subgroups (HR 1.13, 95% CI 0.97–1.33, *P* = 0.110) (Fig. 3). However, the Asian subgroup showed that the trends of PFS benefit for GNP.

**Figure 4:**
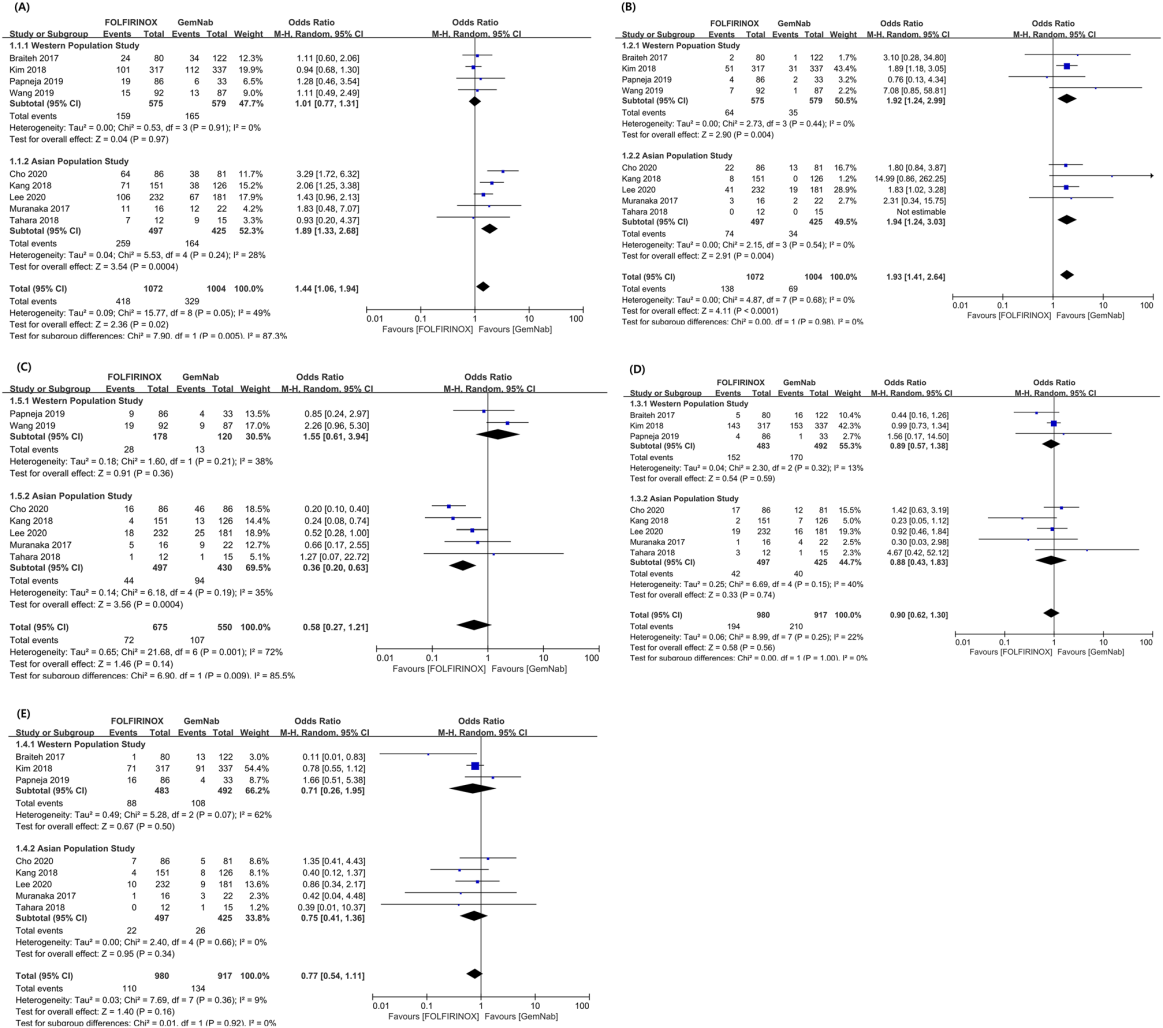
The pooled results of chemotherapy-related toxicities between FOLFIRINOX and GNP. (**A**) For the rate of neutropenia, the occurrence was significantly higher in FOLFIRINOX group than in GNP group (OR 1.44, 95% CI 1.06–1.94, *P* = 0.020), (**B**) for febrile neutropenia, the occurrence was also significantly higher in FOLFIRINOX group than in GNP group (OR 1.93, 95% CI 1.41–2.64, *P* < 0001), (**C**) for peripheral neuropathy, there was no significant difference between the two groups (OR 0.58, 95% CI 0.27–1.21, *P* = 0.140), (**D**) for anemia, there was no significant difference between the two groups (OR 0.90, 95% CI 0.62–1.30, *P* = 0.560), (**E**) for thrombocytopenia, there was no significant difference between the two groups (OR 0.77, 95% CI 0.54–1.11, *P* = 0.160).

The secondary outcomes were the chemotherapy-related adverse effects, including neutropenia, febrile neutropenia, anemia, thrombocytopenia and peripheral neuropathy. The rate of neutropenia development was significantly higher in FOLFIRINOX group than in GNP group (odds ratio [OR] 1.44, 95% CI 1.06–1.94, *P* = 0.020), and it showed a significant heterogeneity (*P* = 0.050, I^2^ = 49.0%). However, when performing the subgroup analysis according to the Western and Asian subgroups, the OR for the neutropenia turned out to be insignificant in the Western subgroup, but it was even higher in FOLFIRINOX of the Asian subgroup (OR 1.01, 95% CI 0.77–1.31, *P* = 0.970 in Western subgroup; OR 1.89, 95% CI 1.33–2.68, *P* < 0.001 in Asian subgroup), and the heterogeneity turned out to be insignificant within the each subgroup (*P* = 0.910, I^2^ = 0% in Western subgroup; *P* = 0.240, I^2^ = 28% in Asian subgroup) (Fig. 4A). For the febrile neutropenia, the OR was also significantly higher in FOLFIRINOX group than in GNP group (OR 1.93, 95% CI 1.41–2.64, *P* < 0.001), which was consistent with the results for neutropenia. However, within each subgroup, occurrence of febrile neutropenia was still significantly higher in FOLFIRINOX (OR 1.92, 95% CI 1.24–2.99, *P* = 0.004 in Western subgroup; OR 1.94, 95% CI 1.24–3.03, *P* = 0.004 in Asian subgroup) (Fig. 4B).

The rate of peripheral neuropathy development was not significantly different between the two groups (OR 0.58, 95% CI 0.27–1.21, *P* = 0.140), and there was a significant heterogeneity (*P* = 0.001, I^2^ = 72%). However, when performing the subgroup analysis, the occurrence rate of peripheral neuropathy in the Asian subgroup was significantly higher in GNP group than in FOLFIRINOX group. Meanwhile, the Western subgroup showed an opposite result, in that, although it did not reach the statistical significance, the rate was higher in FOLFIRINOX group (OR 1.55, 95% CI 0.61–3.94, *P* = 0.360 in Western subgroup; OR 0.36, 95% CI 0.20–0.63, *P* < 0.001 in Asian subgroup) (Fig. 4C). In terms of anemia & thrombocytopenia, there were no significant differences between the two groups (Fig. 4D,E).

There was no possible funnel asymmetry in the analyses of survival outcomes (Supplementary Fig. S4). The funnel plots of chemotherapy-related toxic events did not show any asymmetry, while the analyses of febrile neutropenia showed a slight asymmetry with possible lack of negative effect studies (Supplementary Fig. S5).

## Discussion

This meta-analysis compared the survival outcomes between FOLFIRINOX and GNP as first-line chemotherapy in patients with metastatic PCs. The results indicated that the OS and PFS were not significantly different between FOLFIRINOX and GNP. However, when performing subgroup analyses according to the Western and Asian subgroups, the clinical efficacies turned out to be different between these two regimens; pooled results of the Western subgroup showed better survival outcomes for FOLFIRINOX, whereas the those of the Asian subgroup showed better survival outcomes for GNP. The patients treated with FOLFIRINOX were younger and showed a better performance status in the Western subgroup than the Asian subgroup. Thus, when interpreting the survival status of FOLFIRINOX in the Western subgroup, better performance status should also be included. Meanwhile, better overall survival of GNP in the Asian subgroup was highlighted even though the performance status was not significantly different between the two chemotherapeutic regimens.

Regarding the chemotherapy-related adverse effects, the occurrence of febrile neutropenia was significantly higher in FOLFIRINOX than in GNP. Meanwhile, the occurrence of peripheral neuropathy was not significantly different between the two regimens. When performing subgroup analyses, the occurrence of febrile neutropenia was significantly higher in FOLFIRINOX at both the Western and Asian subgroups; however, peripheral neuropathy turned out significantly higher only in GNP of the Asian subgroup while there were still no significant differences between the two regimens in the Western subgroup. Furthermore, the superior survival of GNP in the Asian subgroup was supported by subset data of the MPACT trial, which demonstrated that the development of peripheral neuropathy was associated significantly with improved survival^32^, because our meta-analysis identified that the Asian subgroup showed a higher peripheral neuropathy in GNP compared with that in the Western subgroup. Therefore, pharmacoethnicity could be one of considering factors when deciding on a frontline chemotherapeutic regimen for patients with metastatic PCs between FOLFIRINOX and GNP.

Pharmacoethnicity has become an important factor in cancer therapeutics^7–10^. The clinical evidence for linking ethnicity and genetic polymorphisms, associated with disease-specific occurrences or cancer drugs sensitivity, have accumulated rapidly^8,10,11,33,34^. As with the advances in genomics, individualized treatments have been studied for various cancers. Germline *BRCA* mutations (gBRCAm) have a favorable prognosis and high sensitivity to platinum-based chemotherapeutics^34,35^. Moreover, the PARP inhibitor, Olaparib, has also shown clinical efficacy in metastatic PC patients with a gBRCAm^36^. Therefore, FOLFIRINOX including oxaliplatin may be a preferred first-line chemotherapy regimen for patients with gBRCAm. However, mutations in the *BRCA* genes have ethnic and geographic heterogeneities, which has been well demonstrated in breast and ovarian cancer^37^. Recently, these differences also have been demonstrated in metastatic PCs; Golan et al*.* reported that the prevalence of gBRCAm was 7.5% in Caucasian and 5.0% in Asian patients. With regard to geographic variability, the highest gBRCAm prevalence was 12.8% in the United States, whereas in South Korea, it was 5.1%^38^. Therefore, this could be a plausible explanation for the favorable survival outcome of FOLFIRINOX over GNP in the Western subgroup. Furthermore, this favorable clinical effect of FOLFIRINOX over GNP in the United States has been recently published by Perri et al., although this study involved locally advanced PCs^39^.

The open access PC dataset of The Cancer Genome Atlas and Queensland Centre for Medical Genomics was analyzed using the cBioportal (http://www.cbioportal.org)^40^, and the ethnic differences of other DNA damage response and repair (DDR) genes mutations were also identified (Supplementary Table S3, S4). Furthermore, the safety or efficacy of anticancer drugs has been shown to have significant correlation with genetic polymorphisms; *UGT1A1* for irinotecan, *DPYD* for fluoropyrimidines, *TPMT* for cisplatin, *CYP3A4, CYP3A5* for taxanes^41^. Irinotecan-induced severe toxicities also have been reported to be related to *ABCB1* polymorphism^42^, which were identified to be higher in Asians^43^. With respect to gemcitabine, the reduced activities of the CDA enzymes by a *CDA* polymorphism (*CDA* 208G > A) may increase the plasma levels of gemcitabine, thereby incurring severe adverse reactions^44^. The *CDA* variant also showed ethnic differences and have been reported to be higher in Asians^45^. As for the aforementioned ethnic differences linked with genes mutations, including gBRCAm, some genetic signatures that were related to FOLFIRINOX or GNP might also have ethnic or geographic differences, which could be another plausible explanation for the survival differences between the Asian and Western subgroups. Therefore, further research is needed to discover specific genetic signatures that are related to the drug susceptibilities of FOLFIRINOX or GNP. If the specific genetic signatures can be found in the future, they can replace the existing one-size-fits-all approach with patient-tailored medicine that can enhance the effectiveness and reduce the toxicities of chemotherapy.

When deciding on the first line chemotherapeutic regimen for patients with metastatic PCs, many factors, including the patient’s baseline characteristics, such as age, performance statuses, and comorbidities, and the clinical policies implemented in each country, such as insurance coverage and drug availability, need to be considered. Furthermore, given that chemotherapy-induced toxicity could lead to early treatment termination, they are also an important factor in terms of determining the type of chemotherapeutic regimen used. The combination regimen of FOLFIRINOX is well known for a higher risk of neutropenia, whereas GNP has a higher risk for neuropathy. These different toxic profiles were also shown in this meta-analysis; adverse events were more intense in the Asian subgroup than in the Western subgroup. These different toxic profiles might be one of the plausible explanations for the different survival outcomes.

There were several limitations in our meta-analyses. First of all, the enrolled studies were all retrospective studies. However, this is inevitable because RCTs regarding this topic have not been conducted so far. Second, the implementation of second-line chemotherapy was not taken into consideration although it might have influenced clinical outcomes. However, the pooled results of PFS, which reflected the clinical effect of first-line chemotherapy, showed the trends of survival benefit of GNP in Asian subgroups. Finally, Western countries are usually composed of many different ethnicities, such as Caucasians, African American, and Hispanics; thereby, the effectiveness of treatment may differ even within Western countries. These findings can be found in the MPACT trial; the effect sizes of palliative chemotherapy were different across regions: Australia, Eastern Europe, Western Europe, and North America^3,4^. In contrast, Asian countries are a relatively homogeneous population than the Western countries; therefore, the favorable outcomes of GNP in the Asian subgroup would have better clinical implications when deciding on the chemotherapeutic regimen for Asian or Asian-heritage patients across the world. However, this study could not discriminate the detailed effects of each ethnic group.

In conclusion, the strength of this meta-analysis is that it is the first meta-analysis to compare the clinical outcomes based on the ethnic subgroups between FOLFIRINOX and GNP as first-line chemotherapeutic regimens in metastatic PCs. Although the survival outcomes were not significantly different between two regimens, the Western subgroup showed better survival of FOLFIRINOX over GNP whereas Asian subgroup showed better survival of GNP over FOLFIRINOX. Therefore, the pharmacoethnicity could be a considering factor for deciding which regimen has to be implemented first for the patients with metastatic PC. Furthermore, future translational research to find out ethnicity-specific genetic polymorphisms as well as unique genetic subsets for defining drug susceptibilities are needed to reach the ultimate goals of personalized cancer therapy.

## Supplementary Information

Supplementary Tables.

Supplementary Figures.
