# Supplementary material for: Pharmacoethnicity of FOLFIRINOX versus gemcitabine plus nab-paclitaxel in metastatic pancreatic cancer: a systematic review and meta-analysis
Source: Sci Rep. 2021 Oct 11;11:20152. doi: 10.1038/s41598-021-99647-5 (PMC8505398; doi:10.1038/s41598-021-99647-5)
Supplement: Supplementary file 1 — Supplementary Tables. [file 41598_2021_99647_MOESM1_ESM.docx]

**Supplemental Table**

**Table S1. Electronic search criteria for systematic review of literature**

| Database | Search criteria | Date |
| --- | --- | --- |
| PubMed | (("pancreas"[Mesh] OR pancrea*[TIAB]) AND ("neoplasms"[Mesh] OR neoplasm*[TIAB] OR cancer*[TIAB] OR "adenocarcinoma"[Mesh] OR adenocarcinoma[TIAB]) AND ("neoplasm metastasis"[Mesh] OR metasta*[TIAB]))  AND  ("carcinoma, pancreatic ductal/drug therapy"[Mesh] OR "pancreatic neoplasms/drug therapy"[Mesh] OR chemo*[TIAB] OR "therapeutics"[Mesh] OR therapy[TIAB] OR treatment[TIAB])  NOT ("case reports" [publication type] OR "review" [publication type]) AND ("gemcitabine"[Supplementary Concept] or gemcitabine[TIAB]) | From 1 Jan 2009  to 30 March 2020 |
| EMBASE | ('pancreas cancer'/exp OR (pancrea*:ti,ab AND (cancer:ti,ab OR neoplasm:ti,ab OR adenoca*:ti,ab))) AND ('metastasis'/exp OR metasta*:ti,ab) AND ('cancer chemotherapy'/exp OR chemo*:ti,ab OR treatment:ti,ab OR therapy:ti,ab) NOT ('case report'/it OR 'conference abstract'/it OR 'review'/it) AND ('gemcitabine'/exp OR gemcitabine*:ti,ab) | From 1 Jan 2009  to 30 March 2020 |
| Cochrane library | #1 MeSH descriptor: [Pancreatic Neoplasms] explode all trees  #2 MeSH descriptor: [Chemotherapy] explode all trees  #3 MeSH descriptor: [Neoplasm Metastasis] explode all trees  #4 #1 AND #2 AND #3 | From 1 Jan 2009  to 30 March 2020 |

**Table S2. Main characteristics of the 13 studies in this meta-analysis**

|  | Study | Year | Country | Institution | Design | Study Period | Enroll Criteria | Total Number of patients | Comparison | Patients number enrolled in each comparison regimen | Baseline characteristics | |
| --- | --- | --- | --- | --- | --- | --- | --- | --- | --- | --- | --- | --- |
|  |  |  |  |  |  |  |  |  |  |  | Age, median (range) or mean (SD) | Performance status (number, percent) |
| 1 | Pacheco-Barcia, et al^18^ | 2020 | Canada & Spain | McGill University Health Center, Montreal; Hospital Universitario La Princesa, Madrid | Retrospective  Multicenter, international, case-control study | January 2010 - January 2018 | mPC | 81 | FFX vs. GNP | FFX (n=22) | 55 (43-72) | ECOG 0;  20 (91%) |
|  |  |  |  |  |  |  |  |  |  | GNP (n=59) | 66 (35-81) | ECOG 0:  32 (54%) |
| 2 | Cho IR, et al^19^ | 2020 | South Korea | Severance Hospital, Seoul, | Retrospective, single-institution case-control study using cohort registry | January 2015 -October 2019 | mPC* | 161 | FFX vs. GNP | FFN (n=86) | 54 (30-78) | ECOG 0;  72 (83.7%) |
|  |  |  |  |  |  |  |  |  |  | GNP (n=81) | 65 (42-79) | ECOG 0;  57 (70.4%) |
| 3 | Lee JC, et al^20^ | 2020 | South Korea | Seoul National University bundang Hospital, Seoungnam; National Cancer Center, Goyang | Retrospective, Multicenter,  case-control study using Korean pancreatic cancer registry | July 2001- December 2017 | mPC | 413 | FFX vs. GNP | FFN (n=232) | 60 (53-66) | ECOG 0 &1;  218 (94%) |
|  |  |  |  |  |  |  |  |  |  | GNP (n=181) | 69 (61-74) | ECOG 0 &1;  166 (92%) |
| 4 | Chan KK, et al^21^ | 2020 | Canada | Sunnybrook Odette Cancer Center, Toronto, | Retrospective, Population-based database study using Ontario Cancer Registry & New drug funding program database | April 2015 – March 2017 | mPC* & LAPC | 1130 (mPC 796, LAPC 334) | FFX vs. GNP | FFX  (n=632;  mPC=416, LAPC=216) | 61.34 (9.33) | ECOG 0;  165 (39.66%) |
|  |  |  |  |  |  |  |  |  |  | GNP  (n=498;  mPC=380,  LAPC=118) | 69.07 (8.71) | ECOG 0;  69 (18.16%) |
| 5 | Williet N, et al^22^ | 2019 | France | University Hospital of Saint-Etienne and European George-Pompidou's Hospital, Paris | Retrospective, two center cohort study, propensity matching | June 2015 – June 2018 | mPC | 216 | FFX vs. GNP | FFX (n=49 after propensity matching, n=107 before propensity matching) | 67 (58-71) | ECOG 0;  13 (26.5%); ECOG 1;  31 (63.3%) |
|  |  |  |  |  |  |  |  |  |  | GNP (n=49 after propensity matching, n=109 before propensity matching) | 66 (59-71) | ECOG 0;  13 (26.5%); ECOG 1;  31 (63.3%) |
| 6 | Papneja N, et al.^23^ | 2019 | Canada | Saskatoon Cancer Center, the university of Saskatchewan, Saskatoon | Retrospective, population-database case-control study using the Saskatchewan cancer agency provincial pharmacy database | 2011-2016 | mPC* & LAPC | 119 | FFX vs. GNP | FFX (n=86) | 59 (54-65) | ECOG 0;  23 (27%);  ECOG 1;  60 (70%); |
|  |  |  |  |  |  |  |  |  |  | GNP (n=33) | 64 (58-70) | ECOG 0;  4 (12.1%); ECOG 1;  24 (73%); |
| 7 | Kang J, et al^24^ | 2018 | South Korea | Asan Medical Center, Seoul | Retrospective, case-control study | January 2013-December 2016 | mPC | 308 | FFX vs. GNP | FFX (n=159) | 60 (25-83) | ECOG 0 & 1; 144 (96.6%); |
|  |  |  |  |  |  |  |  |  |  | GNP (n=149) | 62 (36-82) | ECOG 0 & 1; 158 (99.4%); |
| 8 | Kim S, et al^25^ | 2018 | USA | nationwide | Retrospective, cohort study using physician based national wide chart review | April 2015-December 2015 | mPC* | 578 | FFX vs. GNP | FFX (n=270) | 59.03 (9.46) | ECOG 0; 73 (26.98%)  ECOG 1;  (64.48%) |
|  |  |  |  |  |  |  |  |  |  | GNP (n=308) | 64.59 (9.02) | ECOG 0; 22 (7.09%) ECOG 1;  (63.20%) |
| 9 | Tahara J, et al^26^ | 2018 | Japan | Tokyo Women’s Medical University | Retrospective, case-control study | March 2014-Apri 2017 | mPC & LAPC | 27 | FFX vs. GNP | FFP (n=12; mPC=4, LAPC=8) | 62 (50-72) | ECOG 0 & 1; 100% |
|  |  |  |  |  |  |  |  |  |  | GNP (n=15; mPC=7, LAPC=8) | 63 (44-82) | ECOG 0&1;  100% |
| 10 | Muranaka T, et al^27^ | 2017 | Japan | Hokkaido University Hospital, Hokkaido | Retrospective, single institution cohort study | December 2013- September 2015 | mPC & LAPC | 38 | FFX vs. GNP | FFX (n=16) | 63 (49-78) | ECOG 0;  11 (68.8%); ECOG 1;  5(31.2%) |
|  |  |  |  |  |  |  |  |  |  | GNP (n=22) | 66.5 (49-78) | ECOG 0;  7 (31.8%); ECOG 1;  15 (68.2) |
| 11 | Braiteh F, et al^28^ | 2017 | USA | The Navigating Cancer (NC) database, throughout the USA | Retrospective, population-based database study | September 2014-October 2014 | mPC | 202 | FFX vs. GNP | FFN (n=80) | 61.5 | ECOG 0&1;  36 (82%)* |
|  |  |  |  |  |  |  |  |  |  | GNP (n=122) | 69 | ECOG 0&1;  49 (82%)* |
| 12 | Cartwright TH, et al^29^ | 2018 | USA | The US Oncology Network iKnowMed electronic health record (EHR) system, 400 sites of care in 19 states (Midwest, Northeast, South, and West US census regions) | Retrospective, multi-site, observational cohort study using EHR database | April 2013- October 2015 | mPC | 486 | FFX  vs GNP vs gemcitabine | FFX (n=159) | 61 (28-84) | ECOG 0;  19 (11.9%), ECOG 1;  125 (78.6%); ECOG 2;  71 (14.6%) |
|  |  |  |  |  |  |  |  |  |  | GNP (n=225) | 68 (37-86) | ECOG 0;  20 (7.8%), ECOG 1;  176 (69.0%), ECOG 2;  41 (16.1%) |
|  |  |  |  |  |  |  |  |  |  | Gemcitabine (n=72) | 73 (41-90) | ECOG 0;  9 (12.5%), ECOG 1;  40 (55.6%),  ECOG 2;  18 (25%) |
| 13 | Wagn Y, et al^30^ | 2019 | Canada | The British Columbia Cancer Agency, six medical centers, British Columbia | Retrospective, population-based database study | August 2014- January 2016 | mPC & LAPC | 225 | FFX  vs GNP vs gemcitabine | FFX  (n=92;  mPC=55,  LAPC 37) | 60.35 | ECOG 0&1;  86(92.8%) |
|  |  |  |  |  |  |  |  |  |  | GNP  (n=87;  mPC=66, LAPC=21) | 68.3 | ECOG 0&1;  51(58.6%) |
|  |  |  |  |  |  |  |  |  |  | Gemcitabine (n=46;  mPC=33, LAPC=13) | 74.3 | ECOG 0&1;  17(36.9%) |

***Cases of initial recurrences with distant metastases after surgery were included in the group of metastatic pancreatic cancers.**

**Table S3. Ethnic differences of DNA damage response and repair genes mutation in the open access pancreatic cancer dataset of Queensland Centre for Medical Genomics (QCMG) and The Cancer Genome Atlas (TCGA)**

| **DDR genes** | **Pancreatic Adenocarcinoma**  **(QCMG, Nature 2016)** | | **Pancreatic Adenocarcinoma**  **(TCGA, PanCancer Atlas)** | |
| --- | --- | --- | --- | --- |
|  | **White (n=336)** | **Asian (n=18)** | **White (n=161)** | **Asian (n=11)** |
| **MMR** | 4 (1.2%) | n / s | 6 (3.7%) | n / s |
| **NER** | 2 (0.6%) | n / s | 5 (3.2%) | n / s |
| **HR** | 5 (1.5%) | 1 (5.6%) | 3 (1.9%) | n / s |
| **FA** | 9 (2.7%) | 1 (5.6%) | 8 (4.9%) | 1 (9.1%) |
| **Checkpoint** | 16 (4.8%) | n / s | 11 (7%) | n / s |
| **Others** | 9 (2.7%) | n / s | 4 (2.5%) | n / s |
| **Total** | 45 (13.5%) | 2 (11.2%) | 37 (23.2%) | 2 (18.2%) |

**MMR**: *MLH1, MSH2, MSH6, PMS1, PMS2, EPCAM*; **NER**: *ERCC2, ERCC3, ERCC4, ERCC5*; **HR**: *BRCA1, MRE11A, NBN, RAD50, RAD51, RAD51B, RAD51D, RAD52, RAD54L*; **FA**: *BRCA2, BRIP1, FANCA, FANCC, PALB2, RAD51C, BLM*; **Checkpoint**: *ATM, ATR, CHEK1, CHEK2, MDC1*; **Other**: *POLE, MUTYH, PARP1, RECQL4*

Abbreviations: MMR; DNA mismatch repair, NER; Nucleotide excision repair, HR; Homologous Recombination, FA; Fanconi anemia

**Table S4. Ethnic differences of genes mutation related to the anticancer drug metabolism in the open access pancreatic cancer dataset of Queensland Centre for Medical Genomics (QCMG) and The Cancer Genome Atlas (TCGA)**

|  | **Pancreatic Adenocarcinoma**  **(QCMG, Nature 2016)** | | **Pancreatic Adenocarcinoma**  **(TCGA, PanCancer Atlas)** | |
| --- | --- | --- | --- | --- |
|  | **White (n=336)** | **Asian (n=18)** | **White (n=161)** | **Asian (n=11)** |
| ***UGT1A1*** | n / s | n / s | 2 (1.2%) | n / s |
| ***ABCB1*** | 2 (0.6%) | n / s | 6 (3.7%) | 3 (27.3%)* |
| ***DPYD*** | 2 (0.6%) | n / s | 3 (1.9%) | n / s |
| ***ACYP2*** | n / s | n / s | 1 (0.6%) | n / s |
| ***TPMT*** | n / s | n / s | 1 (0.6%) | n / s |
| ***CDA*** | n / s | n / s | 3 (1.9%) | n / s |
| ***CYP3A4*** | 2 (0.6%) | n / s | 5 (3.1%) | 1 (9.1%) |
| ***CYP3A5*** | 1 (0.3%) | n / s | 4 (2.5%) | 2 (18.2%) |
| **Total** | 7 (2.1%) | n / s | 25 (15.5%) | 6 (54.5%) |

*Statistically significant
