# Supplementary material for: Pharmacoethnicity of FOLFIRINOX versus gemcitabine plus nab-paclitaxel in metastatic pancreatic cancer: a systematic review and meta-analysis
Source: Sci Rep. 2021 Oct 11;11:20152. doi: 10.1038/s41598-021-99647-5 (PMC8505398; doi:10.1038/s41598-021-99647-5)
Supplement: Supplementary file 2 — Supplementary Figures. [file 41598_2021_99647_MOESM2_ESM.pptx]

## Slide 1
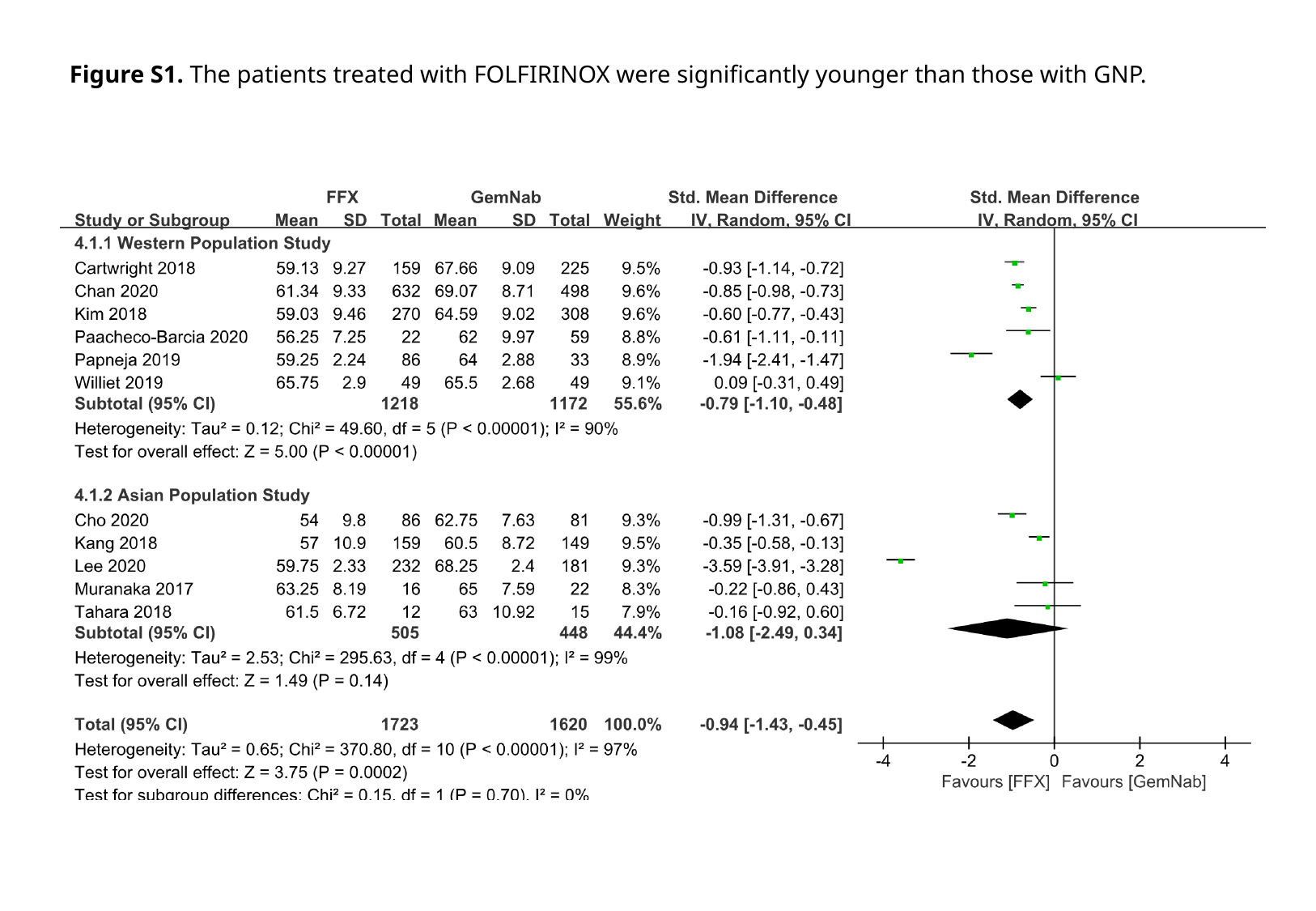

Figure S1. The patients treated with FOLFIRINOX were significantly younger than those with GNP.

## Slide 2
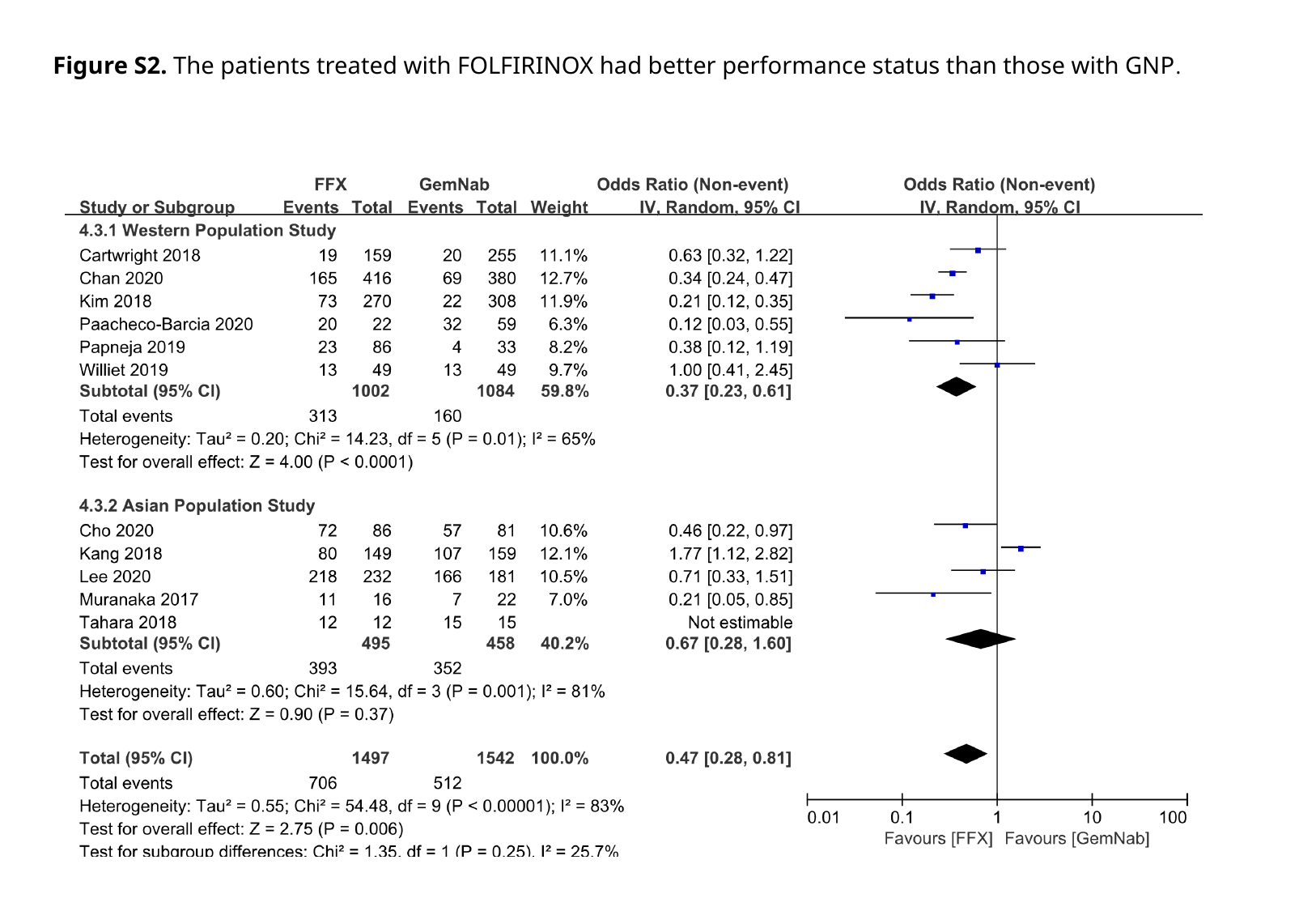

Figure S2. The patients treated with FOLFIRINOX had better performance status than those with GNP.

## Slide 3
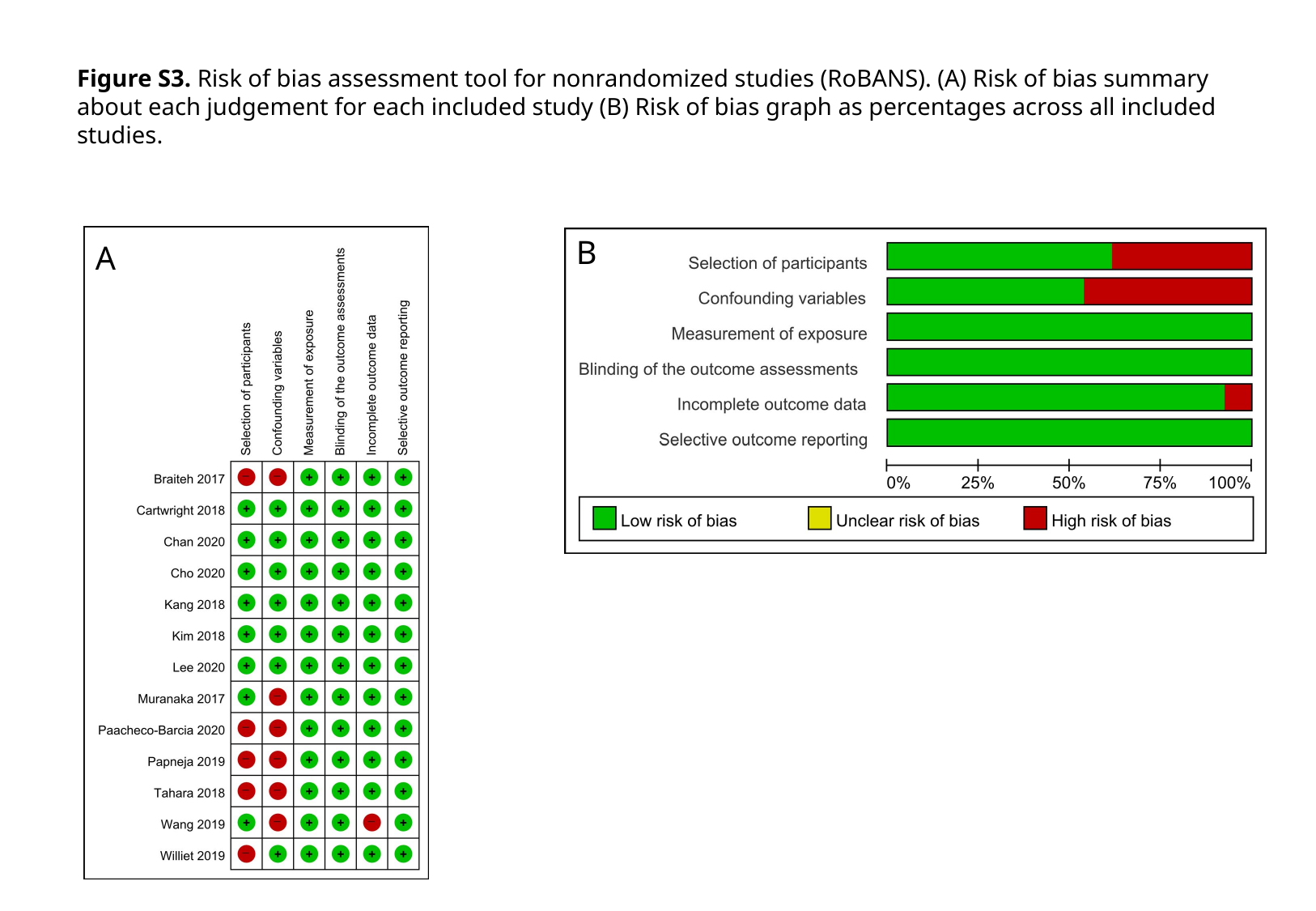

Figure S3. Risk of bias assessment tool for nonrandomized studies (RoBANS). (A) Risk of bias summary about each judgement for each included study (B) Risk of bias graph as percentages across all included studies.
B
A

## Slide 4
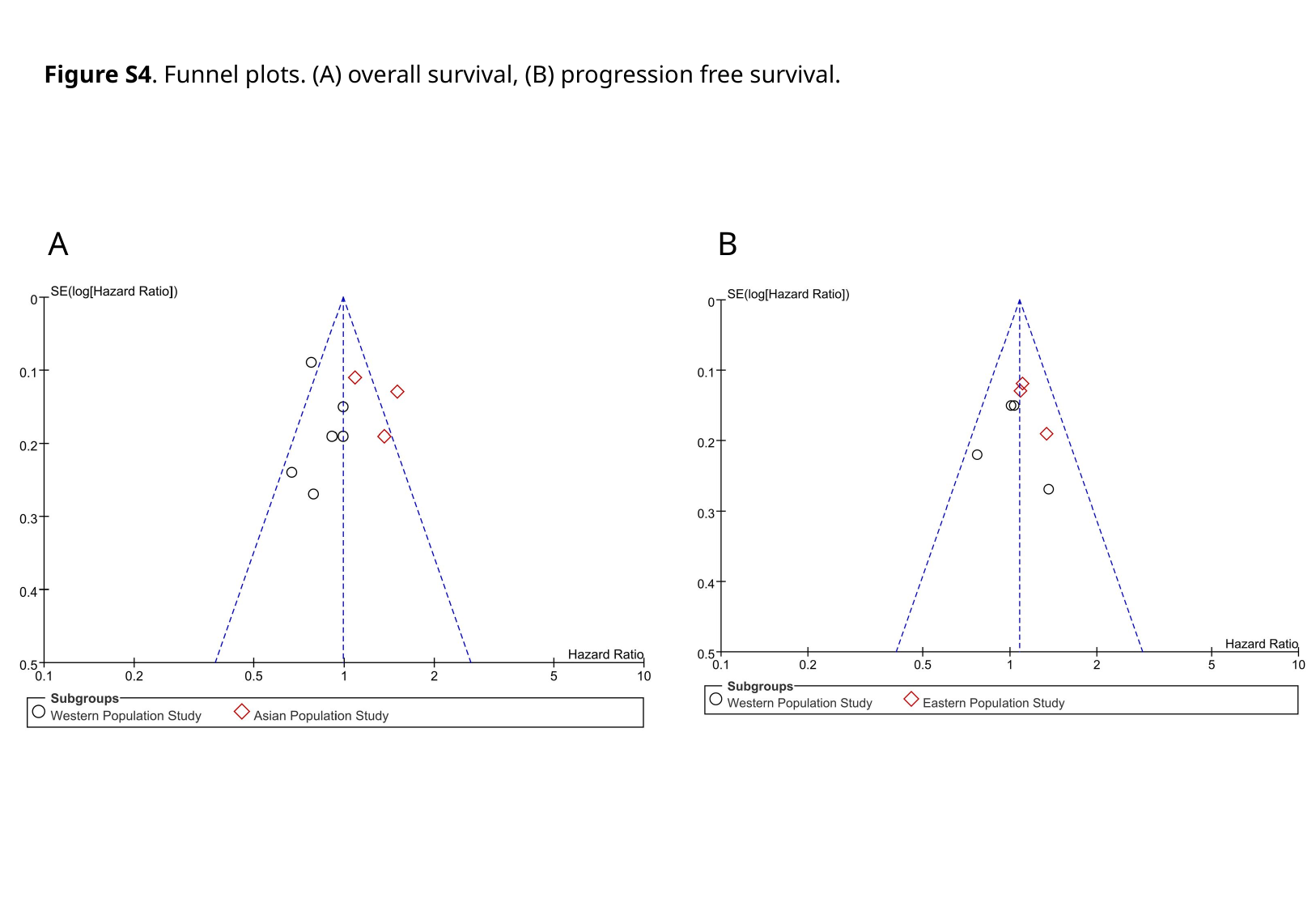

Figure S4. Funnel plots. (A) overall survival, (B) progression free survival.
B
A

## Slide 5
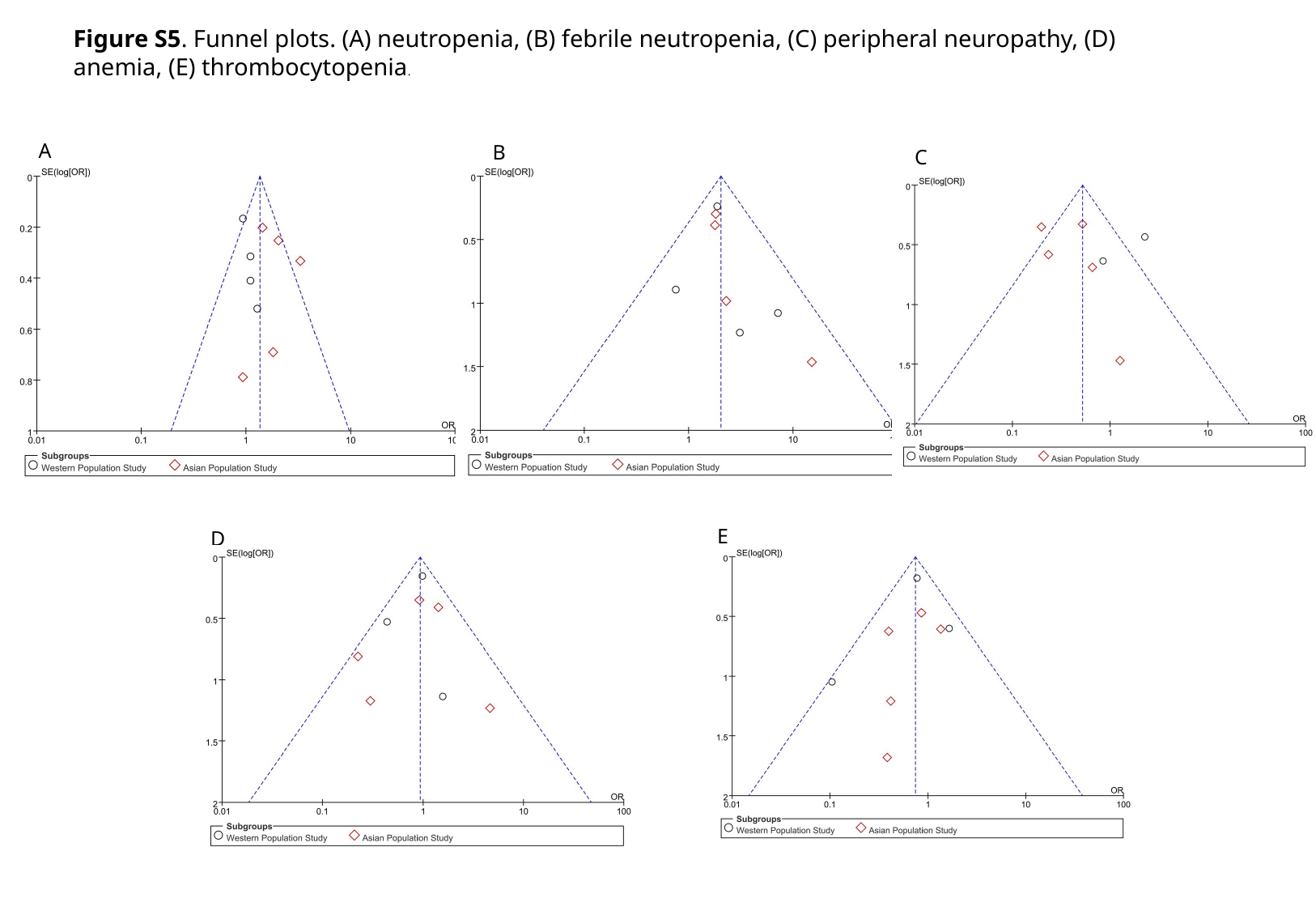

Figure S5. Funnel plots. (A) neutropenia, (B) febrile neutropenia, (C) peripheral neuropathy, (D) anemia, (E) thrombocytopenia.
A
B
C
E
D
